# Parent-child attachment and good behavior habits among Chinese children: Chain mediation effect of parental involvement and psychological Suzhi

**DOI:** 10.1371/journal.pone.0241586

**Published:** 2021-01-06

**Authors:** Xiaoling Mo, Zhi Wang, Jingjin Shao

**Affiliations:** Research Center of Mental Health Education & Faculty of Psychology, Southwest University, Chongqing, China; University of Iowa, UNITED STATES

## Abstract

This study examines the mediation effect of parental involvement and psychological Suzhi between the relationship of parent-child attachment and good behavior habits in Chinese children. The participants comprised 563 children from four Chinese kindergartens (4.41±0.96) whose parents reported measures of parent-child attachment, parental involvement, psychological Suzhi, and good behavior habits in their children. The results indicated that (1) Parental attachment, parental involvement and psychological Suzhi were positively correlated with good behavior habits of young children; (2) Parental involvement and psychological Suzhi mediated the relationship between parent-child attachment and good behavior habits in children separately; (3) Parent-child attachment indirectly affected children’s good behavior habits through the path of “parental involvement and psychological Suzhi.”

## Introduction

Psychologically speaking, behavioral habits are automated behaviors formed by individuals through numerous reuses that are not controlled by consciousness [1]. Good behavior habits refer to the positive behavior patterns that are often displayed in the main activities of an individual, which mainly include four aspects: honesty, practical work, healthy lifestyle, and autonomous learning [2, 3]; Specifically, good habits may include civility, integrity, solidarity, hygiene, diligence, and initiative [3], which has a positive effect on the individual’s life. In China, within the context of quality-oriented education, developing behavior habits has become the focus of young children’s education [4]. Early childhood is a crucial period for the cultivation of behavior habits [5]. Cultivating good behavior habits in this period can help individuals to better adapt to the requirements of society and is essential for individual development. On the contrary, problem behaviors in early childhood not only affect their physical and mental development [6], but also leads to problem behavior subsequently, such as smoking, drinking, etc [7]. That being the case, more attention should be paid to the factors that affect the good behavior habits of young children. Some studies have demonstrated that the parent-child attachment relationship can impacts the formation of individuals’ problem behaviors [8–11]; however, as a positive aspect of behavior development, the impact of parent-child attachment on good behavior habits and the potential mechanisms thereof remain unknown. Thus, in this study, we discuss the influence of the parent-child attachment relationship on children’s good behavior habits and their internal mechanisms; that is, the mediating role of parental involvement and psychological Suzhi in the relationship between parent-child attachment and children’s good behavior habits.

### Parent-child attachment and good behavior habits in children

According to the "ecosystem Theory", the family, as a microsystem, has the most direct and profound impact on individuals [12]. Parent-child attachment is an important aspect of the family environment factor. Empirical studies have confirmed that it can affect the individual problem behavior. For example, some research findings have found that parent-child attachment is not only negatively related to negative emotions such as anxiety and depression in children [13, 14], but also affects individuals’ aggressive behavior and problem Internet use behavior [15, 16]. Similarly, as a positive aspect of individuals’ behavior development, the formation and development of good behavior habits will also be affected by parent-child attachment. In addition, Bowlby’s Attachment Theory defines attachment as an intimate relationship between a child and a particular person and it holds that favorable parent-child attachment is an important factor for children’s growth and development [17, 18], which can provides emotional warmth and social control functions. Emotional warmth enables children’s emotional needs to be met in a timely fashion, which is conducive to children’s acquisition of appropriate emotional management methods. Social control is a regulatory function whereby social members influence others’ behavior in a direct or indirect manner to meet social norms and expectations [19]; specifically, children increase the chance of developing good behavior habits because they do not want to disappoint their parents. Previous studies have found there exist closely correlation between parent-child attachment and good behavior habits, such as good interpersonal skills [20], prosocial behavior [21–23] and positive strategies of emotion regulation [24] and so on, therefore, this present study hypothesized that a positive parent-child attachment relationship is related to fostering good behavior in preschooler.

### The mediating role of psychological Suzhi

Psychological Suzhi is an indigenous Chinese concept, proposed by Zhang and his research team, which has been recognized by Western academia and scholars, and related research has been published in the *Handbook of Positive Psychology in Schools* (Second Edition), an international authoritative reference book [25]. This concept mainly refers to mental qualities that are closely related to human adaptative, developmental, and creative behavior. It is a comprehensive, multi-level system that involves steady, essential, implicit mental quality and explicit adaptive behavior [26]. Furthermore, psychological Suzhi is composed of the following three dimensions: cognitive quality, which refers to individuals’ cognitive process; personality quality, including concepts such as self-esteem, self-confidence, and self-control; and adaptive quality, referring to individuals’ flexibility when faced with a different environment [26, 27]. The Attachment Theory [17] believes that favorable parent-child attachment relationships can promote individuals’ mental health and behavior development; On the contrary, unfavorable parent-child attachment relationships are risk factors for individuals’ growth and development. Empirical Research have found that favorable parent-child attachment have positive significance in improving the individual psychological Suzhi [28], but unfavorable parent-child attachment relationships have negative effects on individuals’ behavior and psychological development, such as problematic Internet use and addiction [16, 29], negative affect [14], social withdrawal behavior [30] and so on. At the same time, previous research found that parent-child attachment can have an impact on individual stress response [31], personality traits [32] and social adaptation [33], which both one part of psychological Suzhi, thus, it can be considered that parent-child attachment plays an essential role in the formation and development of psychological Suzhi.

The theory of psychological Suzhi holds that psychological Suzhi can directly affect individual psychology and behavior [3, 34, 35]. Psychological Suzhi as an individual’s endogenous variable comprises the basis and motivation for individual behavior formation and development [3] and undoubtedly has an important role in the formation and development of individual good behavior. In other words, only individuals with positive psychological qualities can derive and externalize good behavior and then form good behavior habits by numerous reuses, and previous research has found that psychological Suzhi and its dimensions can comprehensively and significantly affect good behavior habits [3].

In summary, this study assumes that psychological Suzhi mediates the relationship between parent-child attachment and good behavior habits of preschoolers.

### The mediating role of parental involvement

Parent-child attachment is a two-way relationship; that is, it can not only affect children’s development, but also parents’ cognition, feelings, and behaviors. A study by Zhang et al. [36] confirm that parent-child attachment can affect parenting style, while Fauth and Thompson [37] affirm that caregivers who have a secure attachment relationship with their children are characterized by sensitivity. Moreover, Rosen and Rothbaum [38] demonstrated that favorable parent-child attachment enables mothers to be more responsive to their children’s needs. Brown, Mangelsdorf, and Neff’s [39] study also shows that secure attachment with children at 13 months predicted greater levels of paternal sensitivity at 3 years. As these articles show, favorable parent-child attachment has a profound effect on parents. In this study, we draw on the views of Lu [40] and Zhang [41] and define parental involvement as “the process whereby parents pour emotions, behaviors, and time into children’s growth and development, in order to promote the comprehensive development of their children.” The parent-child attachment relationship is an emotional link formed between parents and children, which represents the degree of intimacy between them. Thus, better attachment relationship means more intimacy relationship between parents and their children, which enables parents to be more likely to invest resources in their children.

Previous studies have demonstrated that parental involvement is a significant factor influencing individual problem behaviors, such as bullying behaviors [42–45], Shetgiri et al [45] believed that parental involvement play an essential role in reducing problem behaviors of youth. Similarly, as the positive aspect of children’s behavior development, the formation and development of good behaviors can be impacted by parental involvement. And empirical research has also confirmed that parental involvement can significantly promote the growth and development of children in the family [46]. Consequently, it can be considered that parental involvement have an impact on individual good behavior habits.

Summarily, we hypothesized that the influence of parent-child attachment on children’s good behavior habits is achieved through parental involvement.

### Chain mediation effect of parental involvement and psychological Suzhi

As for the relationship between parental involvement and psychological Suzhi or its three dimensions, numerous empirical studies have demonstrated. Grolnick and Benjet [47] and Zhang [41] believe that the importance parents attach to children’s cognitive and emotional development and the investment of additional resources can provide a good environment for children’s cognitive and emotional development, which helps children to acquire more cognitive strategies and enhances their ability to coordinate with the environment, as well as enhancing their psychological Suzhi. Hialdo, Kallemeyn, Leow, Israel, and Lundy’s [48] research indicates that parental involvement has a positive effect on the social and emotional development of children. Hornby [49] believes that a high level of parental involvement can bring about positive changes in children’s values, mental health, and behavior. In her study, Lu [40] suggested that appropriate parental involvement could contribute to the physical and mental health development of children. Therefore, in this study, we believe that parental involvement can affect the psychological Suzhi of young children, and further impact the good behavior of young children through psychological Suzhi.

### Present study

**Fig 1:**
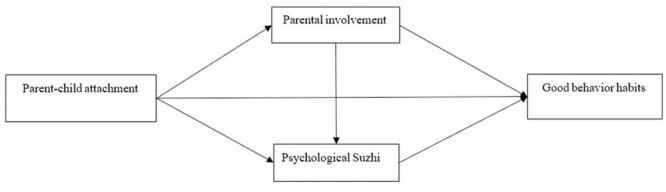
The proposed mediating model.

In summary, the relationship between parent-child attachment and children’s good behavior habits and its underlying mechanism still remain unknown. Consequently, the present study aims to identify the impact of parent-child attachment on good behavior habits in children and the intermediary mechanism of parental involvement and psychological Suzhi. Fig 1 depicts a proposed model. And the proposed research hypotheses are as follows:

H1: There is a significant positive correlation between parent-child attachment, parental involvement and psychological Suzhi, and good behavior habits of young children;
H2, H3: Parental involvement and psychological Suzhi serve a mediating role between parent-child attachment and good behavior in young children;
H4: Parental involvement and psychological Suzhi have a chain mediation effect between parent-child attachment and good behavior habits of young children.

## Methods

### Participants and procedures

The participants comprised 563 young children recruited from four kindergartens in Chongqing, China. Participants were aged 4.41±0.96 years. Of these, there were 290 boys, 273 girls.

This study was approved by the ethics committee for psychological research at the Southwest University. Written informed consent was first obtained from the participating kindergartens and from parents. Before the questionnaires were distributed, verbal informed consent was also obtained from all participants’ parents. All questionnaires in this study were sent to parents by the head teacher and parents answered them accordingly. The criteria and precautions for completing the questionnaire were uniformly stated and the questionnaire was collected on the following day. A total of 600 questionnaires were distributed, 563 children’s parents (155 males, 399 females) finished all questionnaires and their questionnaires were recovered.

### Measures

#### Parent-child attachment

A 18-item questionnaire on parent-child attachment was used in this study(e.g., When you leave your child, your child will be upset; When your child is upset, he or she will seek comfort from you.) [50]. Parents rated on a 5-point Likert scale (1 = completely inconsistent; 5 = completely consistent). Average scores were then calculated to measure the level of parent-child attachment, with higher scores indicating a better attachment relationship between parents and children. The Cronbach’s alpha coefficient in this study was 0.63.

#### Parental involvement

The second part of the Survey on the Education of Rural Parents into Pre-school Children, prepared by Lu [40], was used in our study, and contains 22 items covering three dimensions: cognitive involvement (e.g., telling children a story or reading with young children), emotional involvement (e.g., being able to carefully detect children’s emotional changes), and behavioral involvement (e.g., taking children on trips, visiting places of interest, museums, etc.). Parents assessed items on a 5-point Likert scale (1 = “never”; 5 = “always”). Average scores were then calculated and the higher the scores, the higher the degree of parental involvement. The Cronbach’s alpha coefficient for the present sample was 0.88.

#### Psychological Suzhi

This study used a simplified version of the Child Psychological Suzhi Assessment Questionnaire [3], which has a total of 15 questions, including three dimensions—cognitive quality (e.g., be able to retell the main content of the story or picture more coherently), personality quality (e.g., eager to participate in new games or new events), and adaptive quality (e.g., When they see conflicts between other children, they will take the initiative to persuade them)–which was answered by parents. Questions were rated on a 5-point Likert scale (1 = “completely inconsistent”; 5 = “completely consistent”). We used the average scores of all questions as participants’ final scores. Higher scores indicate a higher level of psychological Suzhi of the participants. The questionnaire has been proved to have good reliability and validity. In this study, the Cronbach’s alpha coefficient was 0.88.

#### Good behavior habits

A 30-item questionnaire on good behavior of young children, developed by Luo [3], was used in this study. This questionnaire contained four dimensions: honesty (e.g., discovering damage caused by others to public property such as tables and chairs and telling the teacher), working hard (e.g., carefully packing up toys after playing), living a healthy life (e.g., participating in sports and activities (e.g., running, shooting, etc.), and studying on their own (e.g., actively reporting their new findings during learning activities). Items were classified into 5 levels (1 = “never”; 5 = “always”) and assessed by parents. The scores were then averaged and the higher the score, the better the behavior habits of participants. In this study, the Cronbach’s alpha coefficient was 0.90.

### Data analyses

SPSS 21.0 and Hayes macro PROCESS in SPSS [51] were used for data analyses. First, descriptive statistics and correlational analyses were conducted to examine whether parent-child attachment was associated with other outcome variables in the expected directions. Second, multiple mediation analyses were conducted to test the mediating role of parental involvement and psychological Suzhi between the relationship of parent-child attachment and good behavior habits. We used 1000 bootstrap samples, and bias were corrected at 95% confidence interval (CI) to calculate the indirect effect of each variable. It indicated that the indirect effect were significant at *p* = 0.05 if the 95% confidence interval didn’t include 0 [52].

## Results

### Common method deviation test

Since the data in this study were all from parents ’self-reports, in order to avoid common methodological deviations, the Harman single factor method was used for statistical control [53], the results showed that there were 21 factors with a characteristic value greater than 1, and the first factor explained a variation of 17.67%, which was much less than the 40% critical value. Therefore, the influence of common method deviation on the results of this study can be excluded.

### Descriptive statistics and correlation analyses

As shown in Table 1, significant correlations were found between parent-child attachment, parental involvement, psychological Suzhi, and good behavior habits of young children. Parents’ involvement was positively related to psychological Suzhi and good behavior habits of young children. Psychological Suzhi was positively related to the good behavior habits of young children.

**Table 1:** Means, standard deviations, and correlations of all variables (n = 563).

	M	SD	1	2	3	4	5	6
1 Parent-child attachment	3.82	.42	-					
2 Parental involvement	3.58	.53	.22***	-				
3 Psychological Suzhi	3.36	.58	.21***	.55***	-			
4 Good behavior habits	3.70	.47	.24***	.57***	.66***	-		
5 Sex	1.48	.50	.01	-.02	.12*	.12*	-	
6 Age	4.41	.96	-.10*	.03	.16	.11*	-.01	-

*p < .05;

**p < .01,

***p < .001.

### Mediation effect test

**Fig 2:**
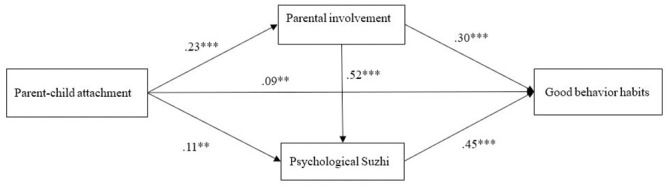
Model of the hypothesized mediator role of parental involvement and psychological Suzhi in the relationship between parent-child attachment and good behavior habits. All the path coefficients were standardized. *p < .05; **p < .01, ***p < .001.

Gender and age were used as control variables in the mediation effect test. The results of the mediation effect analysis are shown in Fig 2 and Table 2. As shown in Fig 2, parent-child attachment significantly predicts parental involvement, psychological Suzhi, and good behavior habits of young children; parents’ involvement significantly predicts psychological Suzhi and good behavior of young children; and psychological Suzhi significantly predicts the good behavior habits of young children. Table 2 shows that the total mediating effect is made up of parent-child attachment through parental involvement, psychological Suzhi, and the mediation chain of parental involvement-psychological Suzhi. The 95% confidence interval corresponding to the three paths is [0.05, 0.12], [0.03, 0.09], [0.04, 0.10], and does not contain 0, indicating that the mediating effect of the three paths is significant. That is, parental involvement and psychological Suzhi play a role in the mediation between parent-child attachment and good behavior habits of young children, while parental involvement and psychological Suzhi play a chain-type mediating role between parent-child attachment and good behavior habits of young children.

**Table 2:** Standardized indirect effects and 95% CIs for the mediational model (n = 563).

Model pathway	Effect	BootSE	BootLLCI	BootULCI
Parent-child attachment→Parental involvement→Good behavior habits	.07	.02	.04	.10
Parent-child attachment→Psychological Suzhi→Good behavior habits	.05	.02	.02	.08
Parent-child attachment→Parental involvement→Psychological Suzhi→Good behavior habits	.05	.01	.03	.08

## Discussion

As anticipated, the results demonstrated a significant positive correlation between parent-child attachment, parental involvement, psychological Suzhi, and children’s good behavior habits. Moreover, the influence of parent-child attachment on children’s good behavior habits is realized by three pathways: the mediation effect of parent-child attachment, the mediation effect of psychological Suzhi and chain-type mediation effect of parental involvement and psychological Suzhi.

The results of correlation analysis verify H1 in this study. Firstly, parent-child attachment has a significant positive correlation with children’s good behavior habits. Parents are one of the most important factors in the family environment system that affects children’s growth and development. While interacting with parents, children subtly acquire parents’ behavior patterns, and secure parent-child attachment means that parents and children interact and communicate more comfortably with each other. Thus, under the influence of the environment and parents’ guidance, children are also more likely to develop good behavior habits. Furthermore, although there are few studies on the direct relationship between parent-child attachment and children’s good behavior habits, previous studies have demonstrated that children with better attachment have lower levels of depression than children with worse attachment and are also less prone to problematic Internet use and other problem behaviors [8, 16]. On the other hand, they scored higher in subjective well-being and school adaptation [54, 55], which facilitates the development of good behavior habits. In addition, previous studies have demonstrated that good parent-child attachment can significantly predict individuals’ social support and self-esteem [54, 56–61], which both closely related with the “internal working models” (Bowlby, 1973), and according to the Attachment Theory, we could know that “internal working models” is the key concept of it and its function lies in the influence on individual psychological and behavioral development. The “internal work model” contains two models: self-representation and other-representation. Self-representation includes the individual’s evaluation and cognition of the present, past, and future of herself; Other-representation refers to the psychological impression that individuals form about important others [62]. Thus, individuals who have established a good attachment relationship with their parents can experience a positive social learning process, which enables them to have positive representations of themselves and others. They believe that they are valuable and capable, and they can feel more social support. They are then able to deal with problems encountered in a suitable way. Consequently, they are more likely to have a good behavior pattern. Secondly, there is a significant positive correlation between parental involvement and children’s good behavior habits, a result that supports the theory of socialization. The theory of socialization holds that parents are promotors of children’s socialization and that parental investment in children can promote the development of children’s self-adjustment capabilities and social adaptation [63]. Previous studies have also shown that parents who are highly involved in their children’s learning and life can enhance their children’s cognitive evaluation ability and social adaptability [55, 64], which can help children to behave better. In addition, Brown et al. [39] found that when parents invest more to children can compensate for the impact of other negative issues toward children, which illustrates the significant impact of parental input on children. Since such parents invest more time and resources into their children, they have more opportunities to guide their children to form good behavior. Thirdly, there is a significant positive correlation between psychological Suzhi and good behavior habits in young children, which is consistent with previous research results [3]. The relationship between psychological Suzhi and mental health illustrates the key role of psychological Suzhi in mental health [65]; that is, individuals with good psychological Suzhi are more adaptable. When experiencing negative life events, they can completely use their own resources and use the correct methods and means to deal with problems. Empirical studies also indicate that individuals with high psychological Suzhi tend to use positive coping strategies [66], and in the process, good behaviors such as practical work and healthy lifestyle form habits through continuous reinforcement.

The results of the mediation effect analysis demonstrate that parental involvement and psychological Suzhi play a mediating role in the relationship of parent-child attachment and good behavior habits. This result support H2 and H3 of this study. Parent-child attachment can not only directly affect the good behavior habits of young children but can also influence good behavior habits through parental involvement and psychological Suzhi. First, the meaning of parental involvement has always been understood as a single parent’s participation in the child’s school education. However, this definition only sees the parent’s behavioral involvement with the child, while overlooking the parent’s cognitive and emotional involvement with the child. The parent-child attachment relationship is an important emotional link between parents and children. It can affect the cognitive, emotional, and behavioral aspects of both children and parents. Therefore, good attachment relationship enables parents to have a closer relationship with their children and therefore to be more likely to invest more resources in them. This view is also supported by empirical research [38, 39], and parents’ high involvement increases the opportunities for interaction between children and parents, which facilitates children’s acquisition of good behavior and continues to strengthen into habits. Secondly, the Attachment Theory [17] holds that individual who have favorable parent-child attachment relationships think they are valuable and worth being loved [67], which can lay a foundation for the development of their cognition, personality, and adaption, and in turn form good psychological Suzhi. Psychological Suzhi, as an individual’s endogenous factor, is a key force for individual development. Individuals with good psychological Suzhi can maintain positive emotions and handle problems appropriately, gradually turning these good behaviors into habits.

The results demonstrate that parental involvement and psychological Suzhi play a chain-mediating role between parent-child attachment and good behavior habits, which confirms H4 proposed in this study. In families where parent-child attachment is secure, children are close to their parents, trust their parents, and are more responsive to their parents’ behavior during interactions with them. This can, in turn, enhance parents’ positive emotional experience and parenting confidence [68]; thus, parents will invest more resources in children. On the one hand, parents’ high involvement enables them to communicate with their children, sense their children’s needs and meet those needs in time, so that children can feel safe and a sense of belonging. Timely communication is also an important guarantee for the development of children’s psychological Suzhi [69]. Additionally, the investment of more resources also provides a positive environment for the development of children’s cognition, personality, and adaptability, which helps children to form a better psychological Suzhi. The cultivation of psychological Suzhi can lay a good psychological foundation for children, which in turn fosters their development of good behavior habits. Individuals with good psychological Suzhi are more likely to develop good behavior patterns in the process of socialization and interpersonal communication. In turn, these good behavior patterns will turn into good behavior habits through continuous strengthening.

While this research has some theoretical and practical significance, it also has the following limitations. First, the data collection method used in this study comprised parental reporting, which may affect the reliability of the data. In future studies, data reliability can be improved by combining parental reporting with teacher reporting. Second, this research uses data as crosscutting data, and cannot control the influence of previous factors on psychological Suzhi and good behavior habits. In the future, longitudinal studies should be conducted to enrich the relevant research results. Last but not least, the formation of good behaviors is the result of multiple factors, such as children themselves, which means future studies may pay attention to the related physiological mechanism of the formation of good behavior habits; and the environment, in “ecosystem theory,” not only parents, but also teachers and peers can have an impact on individuals; however, in this study, we only discussed the joint influence of family factors and individual factors on individuals. Future studies could further add teachers and peer factors to enrich the research regarding the good behavior habits of young children.

This study has discussed the influence of parent-child attachment, parental involvement, and psychological Suzhi on children’s good behavior habits and the internal mechanism thereof and verifies the mediating role of parental involvement and psychological Suzhi between parent-child attachment and children’s good behavior habits. This provides some reference points for parents. First, when parents interact with their children, better attachment relationship and more investment both can promote the development of children, so don’t ignore anyone of them. Second, parents should pay attention to the formation and development of children’s psychological Suzhi and engage in more positive interactions with their children to promote the development of cognition, personality, and adaptability.

## Supporting information

S1 Data (SAV)
